# The effect of different screw-rod design on the anti-rotational torque: a biomechanical comparison of three conventional screw-rod constructs

**DOI:** 10.1186/s12891-017-1683-1

**Published:** 2017-07-28

**Authors:** Zifang Huang, Chongwen Wang, Hengwei Fan, Wenyuan Sui, Xueshi Li, Qifei Wang, Junlin Yang

**Affiliations:** Department of Orthopaedics, The 1st Affiliated Hospital of Sun Yat-sen University, NO.58, Zhongshan Er Road, Guangzhou, Guangdong China

**Keywords:** Anti-rotational torque, Rod-screw construct, Biomechanics

## Abstract

**Background:**

Screw-rod constructs have been widely used to correct spinal deformities, but the effects of different screw-rod systems on anti-rotational torque have not been determined. This study aimed to analyze the biomechanical effect of different rod-screw constructs on anti-rotational torque.

**Methods:**

Three conventional spinal screw-rod systems (Legacy, RF-F-10 and USSII) were used to test the anti-rotational torque in the material test machine. ANOVA was performed to evaluate the anti-rotational capacity of different pedicle screws-rod constructs.

**Results:**

The anti-rotational torque of Legacy group, RF-F-10 group and USSII group were 12.3 ± 1.9 Nm, 6.8 ± 0.4 Nm, and 3.9 ± 0.8 Nm, with a *P* value lower than 0.05. This results indicated that the Legacy screws-rod construct could provide a highest anti-rotation capacity, which is 68% and 210% greater than RF-F-10 screw-rod construct and USSII screw-rod respectively.

**Conclusions:**

The anti-rotational torque may be mainly affected by screw cap and groove design. Our result showed the anti-rotational torque are: Legacy system > RF-F-10 system > USSII system, suggesting that appropriate rod-screw constructs selection in surgery may be vital for anti-rotational torque improvement and preventing derotation correction loss.

## Background

**Fig. 1:**
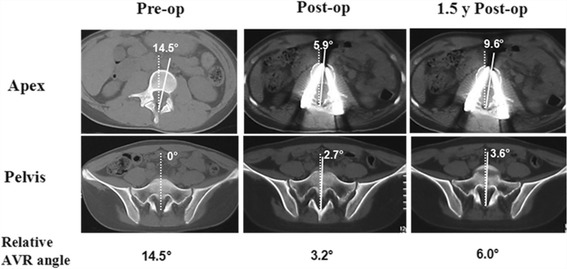
Corrective loss of the relative apical vertebral rotation (*AVR*) angle in a 16-year-old female AIS (Lenke V) with Risser 4. The preoperative relative *AVR angle* was 14.5°, which was calculated from the difference of rotational angles between the pelvis and apical vertebra. The relative *AVR angle* was corrected to 3.2° after surgery, and a 2.8° loss was measured at the 1.5-year follow-up visit

Adolescent idiopathic scoliosis (AIS) is a complex three-dimensional (3D) anomaly of the spine in the coronal, sagittal, and axial planes. Rotational deformity is an important part of AIS and can affect mental health and cause cosmetic defects. Cotrel-Dubousset (CD) instrumentation and rod derotation are excellent techniques for the coronal and sagittal realignment of deformities, but it provides poor rotational improvement for a weak posteromedialization effect [1, 2]. Modern instrumentation systems with pedicle screws are able to provide both real vertebral rotational correction and rib hump correction with the use of direct vertebral rotation (DVR) [3, 4], direct vertebral body derotation (DVBD) [5–8], vertebral coplanar alignment (VCA) [9, 10], or vertebral column manipulator (VCM) techniques [11–13]. Two recent clinical studies [14, 15] reported correction loss in the axial plane despite the use of powerful spinal screw-rod instrumentation, this phenomenon was also found in our clinical practice (Fig. 1). Biological or mechanical factors could both play roles, but to our knowledge, research on this topic is limited.

**Fig. 2:**
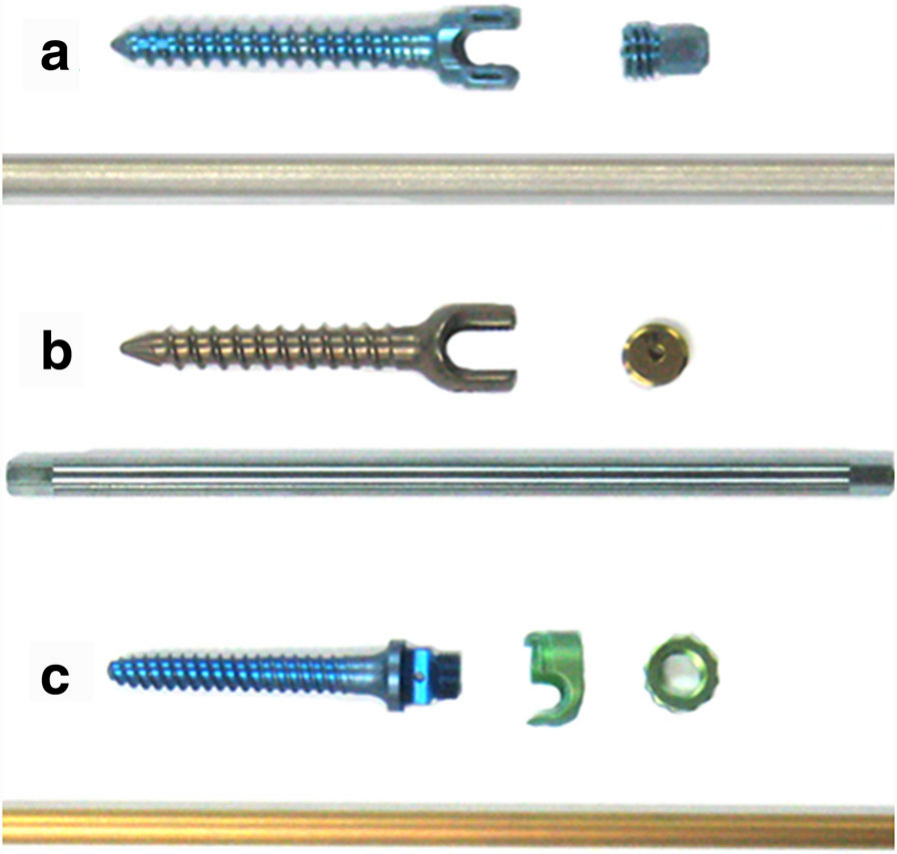
Illustrations of the screw cap and groove characteristics. **a** The Legacy screw includes a “U” groove and a cap with a tip at the *bottom*; **b** The RF-F-10 screw has a “U” groove and a cap with a hole in the *center*; **c** The US II screw consists of an “L” groove and “U” cap

The purpose of this study was to evaluate anti-derotational torque, that is, how much tolerance a screw-rod construct has when twisting the rod in the pedicle screw groove. Here, we assessed the anti-rotational capacities of three different screw-rod constructs (Legacy, RF-F-10, and USSII, as shown in Fig. 2), which were commonly used in China.

## Methods

**Fig. 3:**
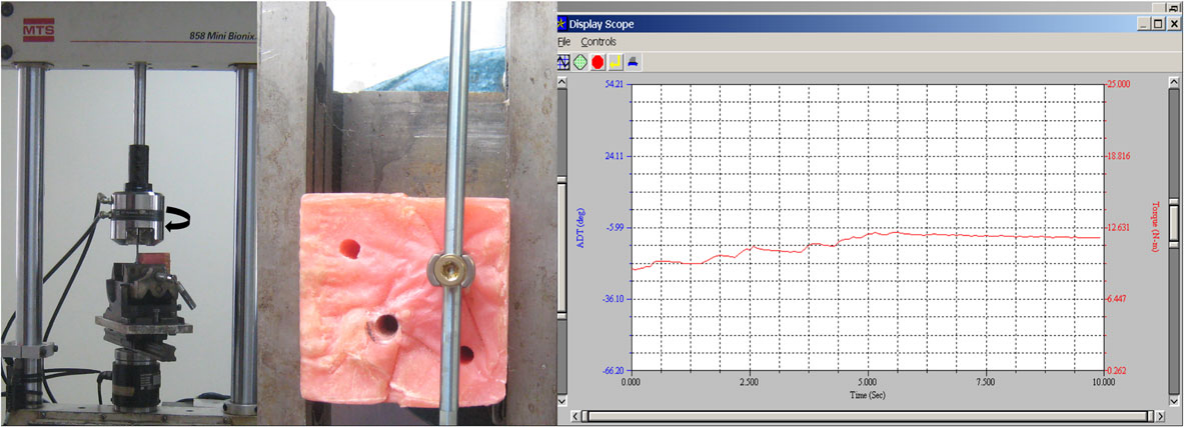
Anti-rotational torque testing. Each rod was twisted (10°/min) in the pedicle screw groove until we were no longer able to measure anti-rotational torque

Three testing groups included different conventional spinal screw-rod fixation systems **(**Legacy, Medtronic Inc., Minneapolis, MN, USA; RF-F-10 screws, Kanghui Inc., China; and USSII, Synthes Inc., West Chester, PA, USA, respectively). Each group comprised seven monoaxial pedicle screws and one round rod (Table 1). One pedicle screw was used from in group in a preliminary study (for testing the machine and adjusting the custom jig before the experiment), and the remaining six pedicle screws were tested in the experiment. Each time, one screw was tightened on the rod with constant torque. The tightening torque is created by twisting off the end cap (11–12.5 Nm) in Legacy group and tightening by 12 Nm in RF-F-10 group and USSII group. Then, part of screw thread was fixed inferiorly with methyl methacrylate to a custom jig in the material test machine (MTS 858 System Inc., Minneapolis, MN, USA), with the free end of rod (3 cm) vertically aligned with the screw axis. Finally, the rod was twisted in the pedicle screw groove at a speed of 10°/min until the torque stopped increasing, and this value was recorded (Fig. 3).

**Table 1:** Instrument parameters

Group	Screw-rod construct	Number of screws	Screw length and diameter (mm)	Rod diameter (mm)
A	Legacy	7	6.5 × 50	5.5
B	USSII	7	6.0 × 45	5.5
C	RF-F-10	7	6.0 × 45	5.5

### Statistical analysis

Statistical comparisons were was carried out using SPSS 17 software (SPSS Inc., Chicago, IL, USA). The anti-rotational torque of three groups are presented as mean ± S.D. Analyses of variance (ANOVAs) were performed to evaluate the posthoc anti-rotational capacities of different pedicle screw-rod constructs. *P*-values <0.05 were considered significant.

## Results

**Fig. 4:**
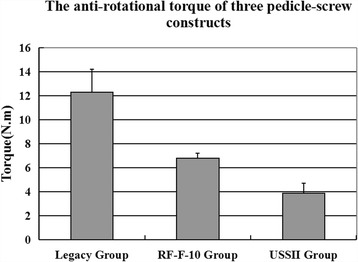
The anti-rotational torques of three conventional screw-rod constructs (*P* < 0.05 among the three groups)

The mean anti-rotational torques of Legacy group, RF-F-10 group and USSII group were 12.3 ± 1.9 Nm, 6.8 ± 0.4 Nm, and 3.9 ± 0.8 Nm respectively, which were significantly different (*P* < 0.01). Posthoc testing comparing pairs of groups revealed that all were statistically different (all *P* < 0.01). The anti-rotational torque of the Legacy screw-rod construct was larger than that of RF-F-10, and the USSII screw-rod construct was the lowest (Fig. 4).

## Discussion

Adolescent idiopathic scoliosis (AIS) is a complex 3D anomaly of the spine. Deformity in a single plane does not develop in isolation; rather, it is dependent on the co-development of curvature, translation, and rotation in other planes [16–18]. Curve progression, secondary thoracic cage deformity, and rib hump are always associated with spinal rotational deformity [19–22]; therefore, vertebral derotation is an important consideration when correcting AIS.

A good 3D correction for scoliosis requires both spinal instrumentation systems and corrective techniques. In the early 1980s, Cotrel-Dubousset (CD) instrumentation with rod derotation was introduced to enable 3D scoliosis correction surgery. However, recent reports suggest that rotational correction is variable (<25%) [23]. Compared to hooks, pedicle screw fixation provides better three-column fixation and 3D correction, which significantly improves the correction rates in the coronal and sagittal planes, particularly for rotational correction of the axial plane Lee et al. (2004), Gabriel et al. (2008), and Huang et al. [3, 9, 11] reported 42.5%, 56%, and 55.2% apical derotations by DVR, VCA, and VCM combined with segmental pedicle instrumentation in AIS, respectively. All three of these techniques also reduce the rib hump, in some cases eliminating the need for a thoracoplasty. This result had been substantiated in other studies [3, 10, 13]. However, Fu et al. [14] reported a recent study of vertebral rotation correction in AIS treated with four different techniques and anchors in which the patients were evaluated by the RAml method on computed tomography scan after 2 years. The authors found that rotation losses in the hook, wire, screw, and anterior groups were 20, 19.4, 17.1, and 12%, respectively. A similar result was described by Cui et al. [15], who reported a mean 2.1° correction loss of apical vertebral rotation angle 2 years after 27 AIS patients were treated with segmental pedicle screw rod constructs. However, to our knowledge, the difference in the anti-rotational capacities of different screw-rod constructs has not been previously described.

In this study, we biomechanically tested the anti-rotational torque of three different pedicle screws-rod constructs. The Legacy screw-rod construct provided the best anti-rotation capacity compared with the RF-F-10 and USSII screw-rod constructs by 68% and 210%, respectively. This suggests that the Legacy screw-rod construct can increase derotational power and better sustain potential derotational correction than the other two spinal instrumentation systems. As the same type of round rods are used in all the three, it is suspected that the anti-rotational torque differences may be caused by differences in pedicle screw design, especially in the screw head and cap part. Indeed, the cap of the Legacy screw is considered to be the main reason for its excellent anti-rotational torque.

The biomechanical testing results demonstrated that the Legacy screw-rod construct provides greater capacity for anti-rotational torque compared to the other two systems. This desirable property is likely affected by different screw cap and groove designs; thus, selecting an appropriate screw-rod system is very important for increasing the anti-rotational torque of a screw-rod construct, and derotational correction loss after AIS surgery is thereby reduced. However, the actual anti-rotational capacity of these three constructs need to be confirmed in clinical comparisons.

**Fig. 5:**
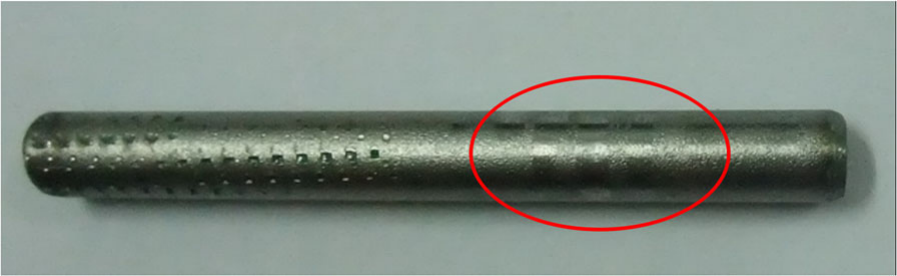
The failure region of RF-F-10 rod was at the groove of screws but not at the junction of rod inside MTS mechanical testing machine regarding the anti-rotational torque

It is important to discuss the limitations of the current study. Only one pedicle screw with straight rod was tested in each simulation, which is both biologically and mechanically different from the situation in which multiple pedicle screws connected with curved rod during surgery. The second limitation was that polyaxial screws test was not considered in this study, although we thought it will more useful for easying the rod placement in clinical practice but not derotation. Furthermore, our findings do not clarify whether the resulting stress concentration by the cap tip on the rod in the Legacy screw-rod construct would cause fatigue failure in the rods (Fig. 5). Thus, further biomechanical evaluations of different systems’ fatigue failure and polyaxial screw’s anti-rotational effect may be needed.

## Conclusions

In summary, we successfully assessed three conventional spinal screw-rod fixations for anti-rotational torque with the MTS 858 Testing System. The results showed that Legacy screws-rod construct can provide better anti-rotational capacity, possibly due to the screw cap and groove designs. The preliminary experimental results demonstrated that appropriate rod-screw construct selection in the clinic is very important for improving anti-rotational torque and preventing derotation correction loss.

## Abbreviations

AIS: Adolescent idiopathic scoliosis
DVBD: Direct vertebral body derotation
DVR: Direct vertebral rotation
VCA: Vertebral coplanar alignment
VCM: Vertebral column manipulator
